# The skate spiracular organ develops from a unique neurogenic placode that is distinct from lateral line placodes

**DOI:** 10.1242/dev.204767

**Published:** 2025-09-29

**Authors:** J. Andrew Gillis, Katharine E. Criswell, Michael A. Palmer, Clare V. H. Baker

**Affiliations:** ^1^ Josephine Bay Paul Center for Comparative Molecular Biology and Evolution, Marine Biological Laboratory, Woods Hole, MA 02543, USA; ^2^ Department of Biology, Saint Francis University, Loretto, PA 15940, USA; ^3^ Department of Physiology, Development and Neuroscience, University of Cambridge, Cambridge CB2 3DY, UK

**Keywords:** Chondrichthyan, *Eya4*, Hair cells, Paratympanic organ, *Sox2*, *Sox3*

## Abstract

The spiracular organ is an epithelial pouch or tube lined with mechanosensory hair cells that is found embedded in the wall of the spiracle in many non-teleost jawed fishes. It is innervated via a branch of the anterior lateral line nerve and usually considered a specialised lateral line organ, despite its presumed function as a proprioceptor for jaw movement. It is homologous to the paratympanic organ: a hair cell-lined epithelial pouch embedded in the wall of the middle ear of birds, alligators and *Sphenodon*. A previous study showed that the chicken paratympanic organ and its afferent neurons originate from a molecularly distinct placode immediately dorsal to the geniculate placode. Here, fate mapping in a cartilaginous fish (little skate, *Leucoraja erinacea*) shows that the spiracular organ derives from a previously unrecognised neurogenic placode immediately dorsal to the geniculate placode that is spatially and molecularly distinct from lateral line placodes. Retrograde labelling of the spiracular organ identified afferent neurons located within the geniculate ganglion, as reported previously for paratympanic organ afferents. These findings support the independence of this unique jawed-vertebrate mechanosensory organ from the lateral line system.

## Teaser

**Summary:** Fate mapping in a cartilaginous fish supports the evolutionary independence from the lateral line system of the mechanosensory spiracular organ, and its homology with the amniote paratympanic organ.

## INTRODUCTION

**Fig. 1.**
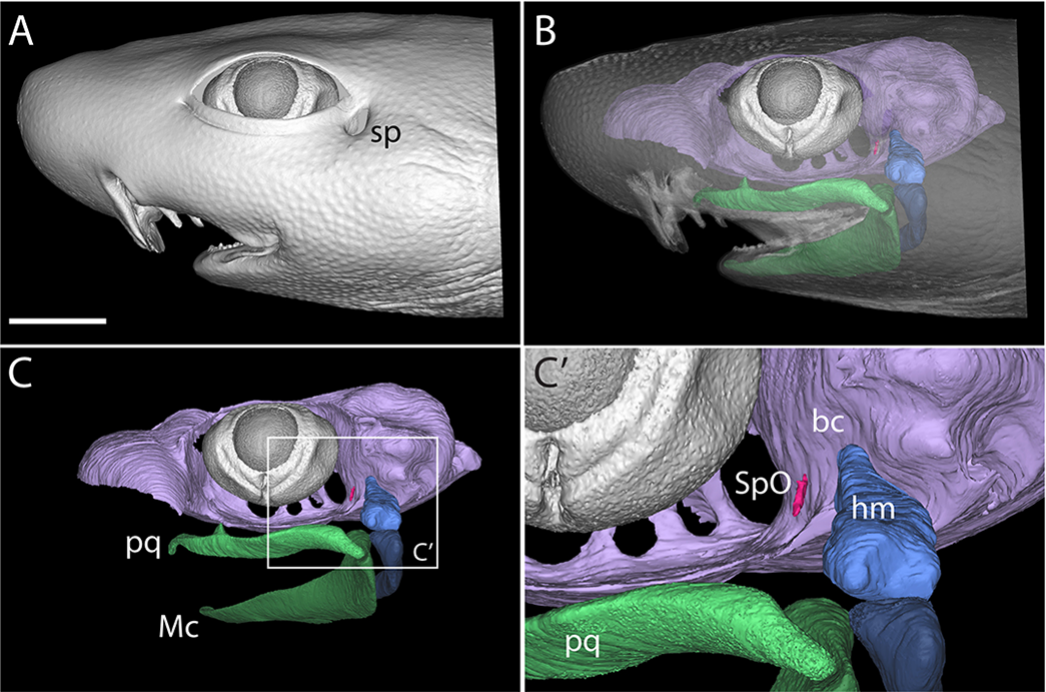
**The spiracular organ of cartilaginous fishes.** MicroCT reconstruction of the head of a pre-hatchling embryonic shark (*Scyliorhinus canicula*). (A) The spiracle (sp) sits behind the eye; it is derived from the first embryonic pharyngeal cleft. (B-C′) The spiracular organ (SpO, magenta) is an epithelial diverticulum embedded in connective tissue between the hyomandibula (hm, blue) and the braincase (bc, lilac). The jaws, consisting of Meckel's cartilage (Mc) and the palatoquadrate (pq), are highlighted in green. Scale bar: 1 cm.

**Fig. 2.**
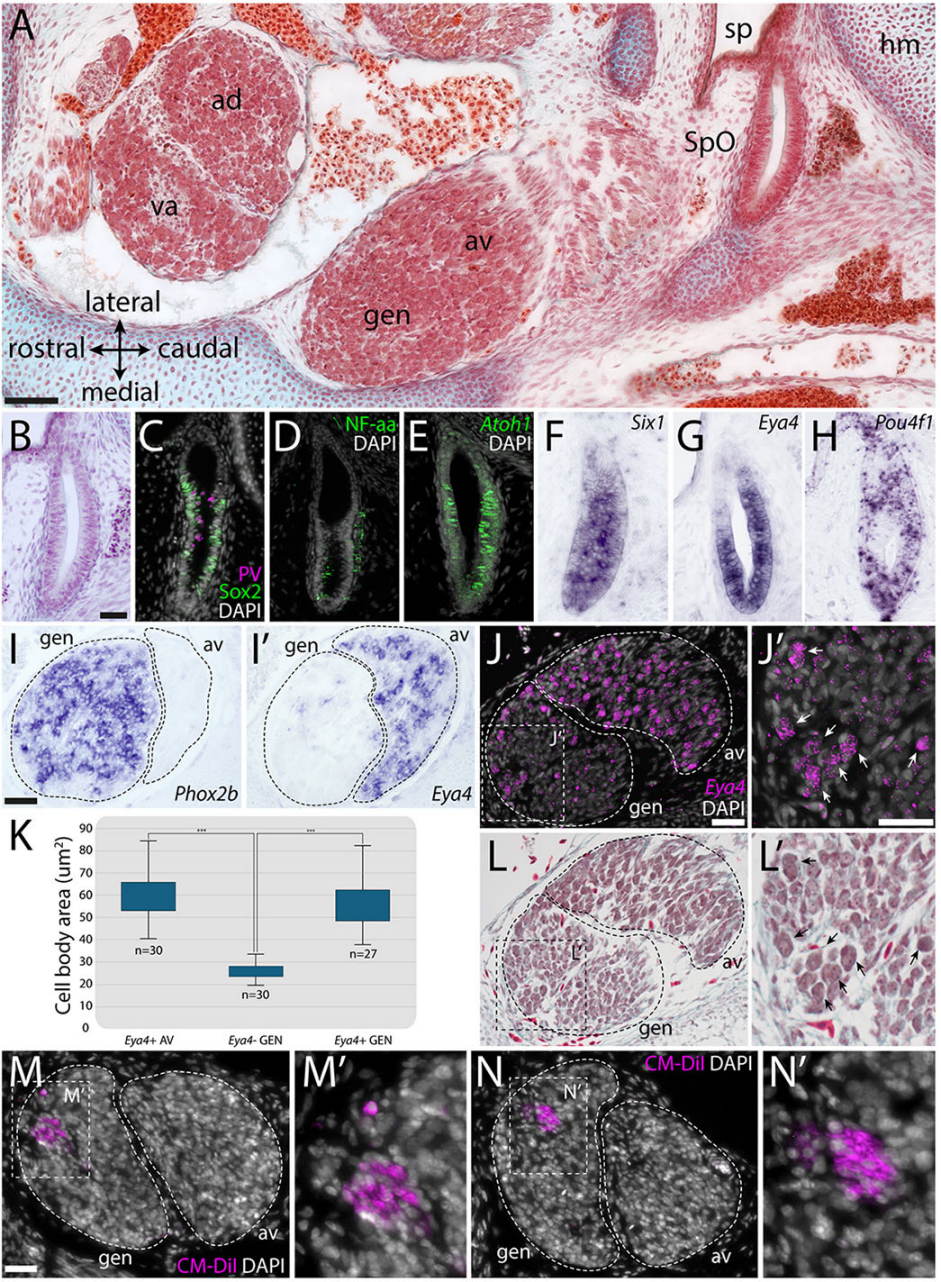
**The spiracular organ and nearby cranial sensory ganglia in late-stage skate embryos.** (A) Masson's trichrome-stained horizontal (frontal) section through the head of a S32 skate embryo at the level of the spiracle, showing the position of the SpO relative to the spiracle (sp), the geniculate ganglion (gen) and the vestibuloacoustic ganglion (va). The geniculate and vestibuloacoustic ganglia form composite ganglia, respectively, with the anteroventral (av) and anterodorsal (ad) lateral line ganglia (LLg). (B) Haematoxylin and Eosin-stained section of the S32 skate SpO. (C,D) At S32, immunofluorescence shows that (C) the SpO expresses the hair cell marker parvalbumin and the supporting cell marker Sox2 in a mutually exclusive pattern, and (D) the sensory epithelium of the SpO is innervated by nerve fibres expressing the neurofilament-associated antigen (3A10). (E-H) *In situ* hybridisation chain reaction (HCR) or *in situ* hybridisation (ISH) on sections shows that the SpO expresses the transcription factor genes (E) *Atoh1*, (F) *Six1*, (G) *Eya4* and (H) *Pou4f1*. (I,I′) ISH on adjacent horizontal (frontal) paraffin sections at S32 shows (I) *Phox2b* expression in the geniculate ganglion and (I′) *Eya4* expression in the anteroventral LLg. *Eya4*-positive cells are also scattered amongst the *Phox2b*-positive neurons in the geniculate ganglion. (J,J′) HCR shows *Eya4* expression in the anteroventral LLg and in scattered cells within the geniculate ganglion (white arrows in J′). (K) Box and whisker plot (boxes show upper and lower quartiles; whiskers show upper and lower extremes) showing that *Eya4*-positive anteroventral LLg neurons (*Eya4*+ AV) are the same size (cell-body area) as *Eya4*-positive cell bodies within the geniculate ganglion (*Eya4*+ GEN), but both *Eya4*-positive cell types are significantly larger than *Eya4*-negative cell bodies in the geniculate ganglion (*Eya4*− GEN). *Eya4*-positive and *Eya4*-negative cell-body areas were measured using FIJI on sections stained post-HCR with Masson's trichrome (see L,L′). For each cell type, ten cell bodies were measured in each of three individuals, except for one individual that had only seven *Eya4*-positive geniculate cells. A Kruskal–Wallis H test indicated a significant difference between the different groups: χ^2^(2)=59.88, *P*<0.001, with a mean rank score of 63 for *Eya4*+ AV (*n*=30), 54.56 for *Eya4*+ GEN (*n*=27) and 15.5 for *Eya4*− GEN (*n*=30). A post-hoc Dunn's test using a Bonferroni corrected alpha of 0.017 indicated that the mean ranks of the following pairs are significantly different: *Eya4*+ AV versus *Eya4*− GEN and *Eya4*− GEN versus *Eya4*+ GEN. (L,L′) The same sections as in J,J′ stained post-HCR with Masson's trichrome. Black arrows in L′ indicate the same *Eya4*-positive geniculate cells indicated by white arrows in J′. (M-N′) Frontal sections through the geniculate/anteroventral lateral line ganglionic complex from two different S32 embryos after retrograde labelling of the SpO with CM-DiI. In both cases, multiple CM-DiI-positive cell bodies are present in the geniculate ganglion. ad, anterodorsal lateral line ganglion; av, anteroventral lateral line ganglion, gen, geniculate ganglion; hm, hyomandibula; sp, spiracle; SpO, spiracular organ; va, vestibuloacoustic ganglion. Scale bars: 50 µm in A; 25 µm in B-H; 25 µm in I-J′,L,L′; 25 µm in M-N′.

The spiracular organ (SpO) is an epithelial diverticulum lined with mechanosensory hair cells embedded in the wall of the spiracle or spiracular chamber (a remnant of the first pharyngeal cleft) in representatives of all groups of extant jawed vertebrates: cartilaginous fishes; non-teleost ray-finned fishes, excluding bichirs; and the lobe-finned lungfishes and coelacanth (for example, Allis, 1889; Agar, 1906; Norris and Hughes, 1920a; Barry and Bennett, 1989; Johnston, 2022). In sharks and skates (elasmobranchs), the SpO is embedded in connective tissue near the articulation between the hyomandibula and the braincase (Barry and Boord, 1984; Barry et al., 1988a; Barry and Bennett, 1989) (Fig. 1, Fig. 2A). Afferent innervation is provided via a branch of the anterior lateral line nerve (Barry and Boord, 1984) and the SpO is usually considered to be a specialised lateral line organ (Barry and Bennett, 1989; Northcutt, 1989). However, SpO afferents project centrally not only to the mechanoreceptive lateral line nucleus and vestibulocerebellum but also, uniquely, to lateral regions of the reticular formation (Barry and Boord, 1984), and they are excited by flexion of the hyomandibula at the cranial joint, which distorts the SpO (Barry et al., 1988b). These findings led to the proposal that the elasmobranch SpO is a proprioceptor for jaw movement (Barry et al., 1988b; Barry and Bennett, 1989). Based on the anatomy of the closed spiracular pouch, the lungfish spiracular organ was also suggested to act as a proprioceptor for jaw and hyoid or opercular movements: it is compressed by the spiracular cartilage when the jaw closes, and extended or compressed when the opercular apparatus moves outwards or inwards, respectively (Bartsch, 1994).

In tetrapods, the spiracular chamber evolved into the middle ear cavity and the hyomandibula evolved into the columella/stapes (Clack, 2002). Birds, alligators and the tuatara (*Sphenodon*) possess a mechanosensory hair cell-containing epithelial pouch, the ‘paratympanic organ’ (PTO), embedded in connective tissue in the wall of the middle ear (Simonetta, 1953; Werner, 1963; Neeser and von Bartheld, 2002; von Bartheld and Giannessi, 2011; Giannessi et al., 2013). Given their similar anatomy and shared association with derivatives of the first pharyngeal cleft (i.e., spiracle, middle ear), as well as PTO-afferent projections to vestibular nuclei and the ventral cerebellum (von Bartheld, 1990), it has long been proposed that the SpO and PTO are homologous (see von Bartheld and Giannessi, 2011; Giannessi et al., 2013). However, at odds with the hypothesis of homology was the apparent embryonic origin of the PTO from the geniculate placode, i.e., the first of the ‘epibranchial placodes’ that form dorsocaudal to each pharyngeal cleft and give rise to viscerosensory neurons in the distal sensory ganglia of cranial nerves VII, IX and X (the geniculate, glossopharyngeal and nodose ganglia, respectively) (see Ladher et al., 2010). Ablation and fate-mapping experiments in chicken embryos showed that ectoderm in the region of the geniculate placode forms the PTO as well as geniculate neurons (Yntema, 1944; D'Amico-Martel and Noden, 1983). A geniculate placode origin was also consistent with nerve-tracing experiments that identified PTO-afferent neurons within the geniculate ganglion and collected in a nearby ‘paratympanic extension’ of the geniculate ganglion (von Bartheld, 1990). Clonal analysis via retroviral lineage-labelling yielded similar results (Satoh and Fekete, 2005).

The conflict between an apparent geniculate placode origin for the PTO and its proposed homology with the spiracular organ was resolved by fate-mapping and other experiments demonstrating that the chicken PTO and its afferent neurons arise from a molecularly distinct placode located immediately dorsal to the geniculate placode (O'Neill et al., 2012). Here, we sought to determine experimentally the embryonic origin of the SpO in a cartilaginous fish, the little skate (*Leucoraja erinacea*).

## RESULTS AND DISCUSSION

At stage (S)32 in the little skate (pre-hatching: around 80 days post-oviposition; Gillis et al., 2022), the SpO is easily identified on histological sections, adjacent to the spiracle and near the hyomandibula (Fig. 2A,B). Just as for the chicken PTO at S34 (embryonic day 8), as well as 3 days earlier at S27 (O'Neill et al., 2012), immunofluorescence reveals expression in the skate SpO of a hair cell marker (Fig. 2C: parvalbumin, a calcium-buffering protein); this is mutually exclusive with the supporting cell marker Sox2 (a SoxB1-class transcription factor) (Fig. 2C; Fig. S1). The SpO sensory epithelium is innervated by nerve fibres expressing a neurofilament-associated antigen (Fig. 2D). *In situ* hybridisation chain reaction (HCR) shows that, like the developing PTO (O'Neill et al., 2012), the SpO expresses *Atoh1* (Fig. 2E), which is essential for hair cell development in bony vertebrates (Bermingham et al., 1999; Millimaki et al., 2007). *In situ* hybridisation reveals SpO expression of *Six1* (Fig. 2F), which is expressed by all neurogenic placodes and their derivatives (see Schlosser, 2014; Moody and LaMantia, 2015), and *Eya4* (Fig. 2G), a conserved marker for otic and lateral line placodes and their derivatives across jawed vertebrates, including the little skate (O'Neill et al., 2007; Modrell et al., 2011a; Gillis et al., 2012; Modrell and Baker, 2012). *Pou4f1* (*Brn3a*) is also expressed in the SpO (Fig. 2H). In the developing chicken PTO, Pou4f1-positive, Islet1-positive neuroblasts are still delaminating from the PTO at S27 (O'Neill et al., 2012). In the little skate, a few *Pou4f1*-positive cells were seen at the caudal edge of the SpO at S32 (Fig. 2H), but cells near the SpO were not immunoreactive for Islet1 or any other neuronal differentiation markers tested (Islet1/2, Tubb3, Elavl3/Elavl4, NeuN; data not shown). Stage 32 (pre-hatching) may be too late to detect delaminating neuroblasts.

In chicken embryos, the cell bodies of PTO-afferent neurons are scattered within the geniculate ganglion and collected in a small separate ganglion, the ‘PTO ganglion’ (von Bartheld, 1990; O'Neill et al., 2012). PTO neurons express the vestibuloacoustic neuron marker Pou4f1, but not the epibranchial placode-derived neuron marker Phox2b, and at least by S35 (embryonic day 9), they are significantly smaller than geniculate neurons (O'Neill et al., 2012). In the little skate at S32, *in situ* hybridisation on adjacent sections for *Phox2b* versus the lateral line/vestibuloacoustic ganglion marker *Eya4* (O'Neill et al., 2007) revealed a few *Eya4*-positive cells scattered amongst the *Phox2b*-positive geniculate neurons, as well as throughout the anteroventral lateral line ganglion, which forms a ganglionic complex with the geniculate ganglion (Fig. 2I,I′). HCR confirmed the presence of *Eya4*-positive cells in the geniculate ganglion (Fig. 2J,J′). We found that the cell bodies of *Eya4*-positive geniculate cells were the same size as *Eya4*-positive cells in the anteroventral lateral line ganglion, but both were significantly larger than *Eya4*-negative geniculate cell bodies (Fig. 2K). This was determined by measuring cell-body area on post-HCR trichrome-stained sections (Fig. 2L,L′). Given that PTO-afferent neurons within the embryonic chicken geniculate ganglion are morphologically distinct and express a vestibuloacoustic neuron marker (O'Neill et al., 2012), we speculate that the morphologically distinct, *Eya4*-positive cells scattered within the skate geniculate ganglion could represent SpO-afferent neurons. We attempted to locate SpO afferents by injecting CM-DiI into the SpO of fixed S32 embryos for retrograde labelling (Fig. S2). Only two out of four embryos showed CM-DiI diffusion all the way to sensory ganglia, but in both, multiple DiI-positive cell bodies were located within the geniculate ganglion (Fig. 2M-N′). These data suggest that, like chicken PTO afferents (O'Neill et al., 2012), at least some skate SpO afferents are found within the geniculate ganglion.

**Fig. 3.**
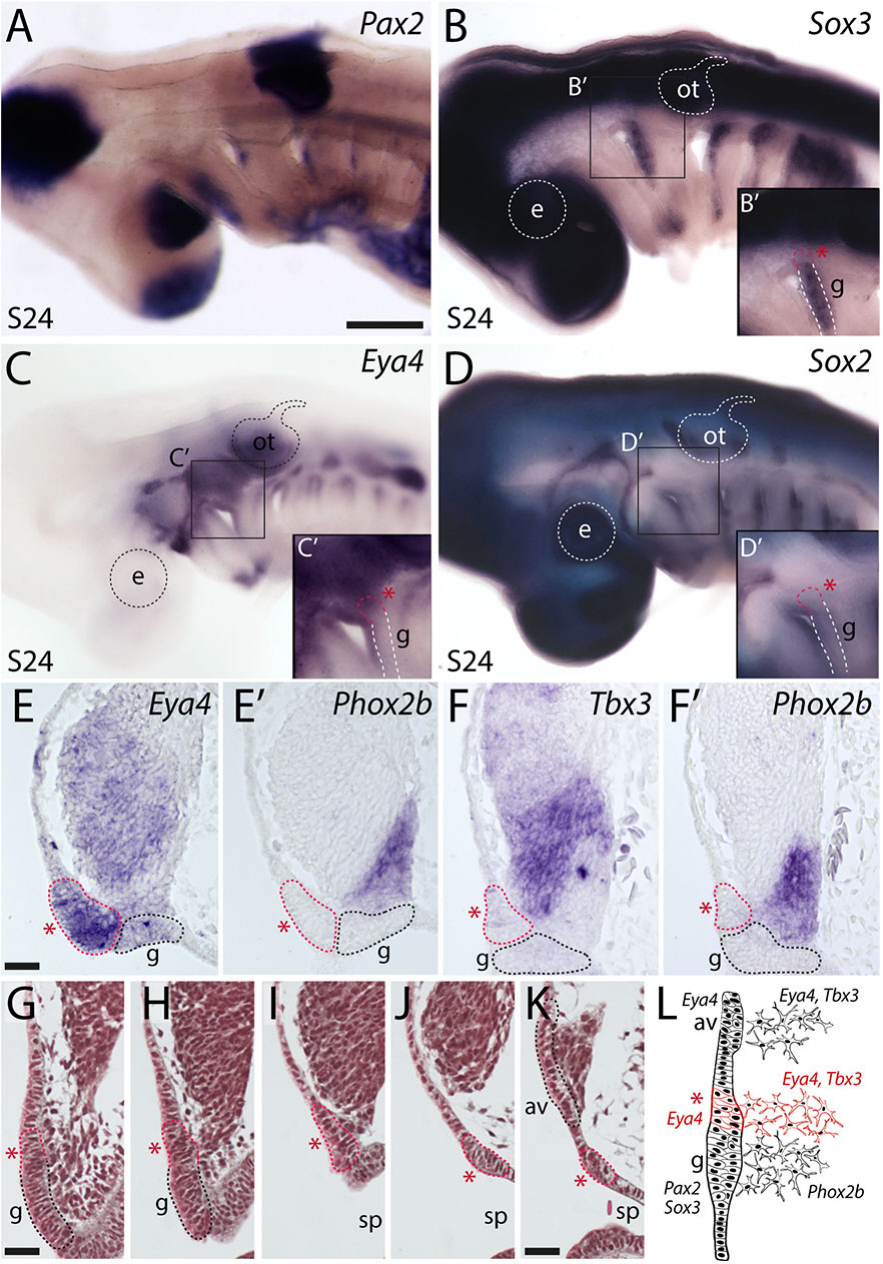
**A putative spiracular organ placode in the skate, immediately dorsal to the geniculate placode.** Given the homology of the amniote PTO and the SpO, the putative SpO placode in skate is expected to lie immediately dorsal to the geniculate (first epibranchial) placode, above the first pharyngeal (spiracular) cleft. (A-D) Whole-mount *in situ* hybridisation at S24 for *Pax2* (A) and *Sox3* (B) identifies the epibranchial placodes dorsocaudal to each pharyngeal cleft. Ectoderm immediately dorsal to the geniculate placode expresses *Eya4* (C) and is distinct from *Sox2*-positive elongating lateral line primordia (D). Insets show higher-power views of the first pharyngeal (spiracular) cleft region. (E,E′) *In situ* hybridisation on adjacent transverse paraffin sections at S24 reveals a domain of *Eya4*-positive placodal ectoderm (red asterisk) and subjacent mesenchyme, presumably neuroblasts, immediately dorsal to the geniculate placode, here identified as a placodal source of *Phox2b*-positive neuroblasts in the ventral region of the adjacent ganglion. (F,F′) This domain of placodal ectoderm (red asterisk) is also a source of *Tbx3*-positive neuroblasts, which emigrate immediately dorsal to *Phox2b*-positive geniculate placode-derived neuroblasts. (G-K) Selected transverse histological sections at S24 in a caudal-to-rostral sequence, including the putative SpO placode (red asterisk and red dotted line in all sections). Successively, the sections show: (G) the geniculate placode (g) with emigrating neuroblasts; (H,I) the putative SpO placode with emigrating neuroblasts lying immediately dorsal to the geniculate placode; (J) a break in the placodal ectoderm; (K) a dorsal neurogenic placode with emigrating neuroblasts, expected to be the neurogenic pole of the anteroventral lateral line placode (av). All these regions of neurogenic placodal ectoderm contribute neuroblasts to the same ganglion. The complete series of histological sections is provided as Movie 1. (L) Schematic illustrating the spatial organisation, relative locations and gene expression of the geniculate, SpO and anteroventral lateral line placodes, and their neuroblasts, at S24. Red asterisk indicates the SpO placode. av, anteroventral lateral line placode; e, eye; g, geniculate placode; ot, otic vesicle; sp, spiracle. Scale bars: 500 µm in A-D; 25 µm in E-K.

We then attempted to identify a putative SpO placode using candidate gene expression and histology. Classical histological studies in embryonic lungfish (Agar, 1906), gars (Landacre and Conger, 1913; Hammarberg, 1937) and shark (Holmgren, 1940) all suggested that the SpO is derived from a primordium that is separate from the lateral line placodes. The PTO placode can be recognised externally in chicken embryos at stage 18 (Hamburger and Hamilton, 1951) as a patch of *Sox2*-positive ectoderm immediately dorsal to the *Pax2*-positive, *Sox3*-positive, *Sox2*-negative geniculate placode, which itself lies dorsocaudal to the first pharyngeal cleft (O'Neill et al., 2012). In the little skate at S24, *Pax2* expression identifies the maturing epibranchial placodes caudal to the dorsal region of each pharyngeal cleft (Fig. 3A), as previously reported in shark (O'Neill et al., 2007). The epibranchial placodes also express *Sox3*, in a broader stripe extending further ventrally than *Pax2* at this stage (Fig. 3B). Contrary to our expectations from bony vertebrates, in which *Sox3* is strongly expressed by lateral line as well as epibranchial placodes (for example, Schlosser and Ahrens, 2004; Modrell et al., 2011b), *Sox3* was not expressed by elongating lateral line primordia in skate (Fig. 3B): these express *Eya4* (Fig. 3C), as previously reported in shark (O'Neill et al., 2007) and skate (Gillis et al., 2012), and *Sox2* (Fig. 3D). Higher-power views of the first pharyngeal (spiracular) cleft region (insets in Fig. 3A-D) revealed a domain of *Eya4*-positive ectoderm immediately dorsal to the geniculate placode and extending slightly rostrally to it, i.e., in a similar position to the chicken PTO placode (O'Neill et al., 2012), but lacking *Sox2* expression, in contrast to lateral line primordia. The absence of *Sox2* also contrasts with the chicken PTO placode, for which *Sox2* is an early marker, maintained throughout PTO development (O'Neill et al., 2012). Skate embryos develop slowly, and it is possible that we missed transient *Sox2* expression. However, we examined multiple embryos and could not detect *Sox2* expression here at epibranchial placode stages (although at S32, Sox2 is expressed in the SpO epithelium; Fig. 2C).

*In situ* hybridisation on adjacent transverse sections at the level of the second pharyngeal (hyoid) arch showed that the *Eya4* expression domain seen in whole mount (Fig. 3C) includes placodal (thickened) ectoderm (Fig. 3E′) lying immediately dorsal to the *Eya4*-negative geniculate placode, which generates *Phox2b*-positive neurons in the ventralmost wedge of the large adjacent ganglion (Fig. 3E′). The *Eya4*-positive putative SpO placode also seems to be the source of a tranche of strongly *Tbx3*-positive cells in the ganglion (Fig. 3F), immediately dorsal to the *Phox2b*-positive neuroblasts emigrating from the geniculate placode (Fig. 3F′). Serial histology at S24 confirmed the existence of a domain of neurogenic placodal ectoderm extending dorsally from the geniculate placode above the first pharyngeal (spiracular) cleft, separated by thin non-placodal ectoderm from a discrete neurogenic placode lying further rostral and dorsal, all three placodes apparently contributing neuroblasts to the same developing ganglion/ganglionic complex (Fig. 3G-K; Movie 1; schematised in Fig. 3L). The strongly *Tbx3*-positive cells medially and the weakly *Tbx3*-positive cells dorsally in the composite ganglion also express *Eya4* (compare with Fig. 3E). The source of the dorsal neuroblasts must be the discrete neurogenic lateral line placode identified by serial histology further rostral and dorsal to the putative SpO placode (Fig. 3K; Movie 1). This is presumably the neurogenic pole of the anteroventral lateral line placode, as the mature composite ganglion comprises the geniculate and anteroventral lateral line ganglia (Fig. 2A,J-L; also see Landacre, 1916; O'Neill et al., 2007). Overall, these molecular and histological data suggest that the putative SpO placode (*Eya4* positive and *Sox2* negative) is both spatially and molecularly distinct from lateral line primordia (*Eya4* positive and *Sox2* positive) in the little skate.

**Fig. 4.**
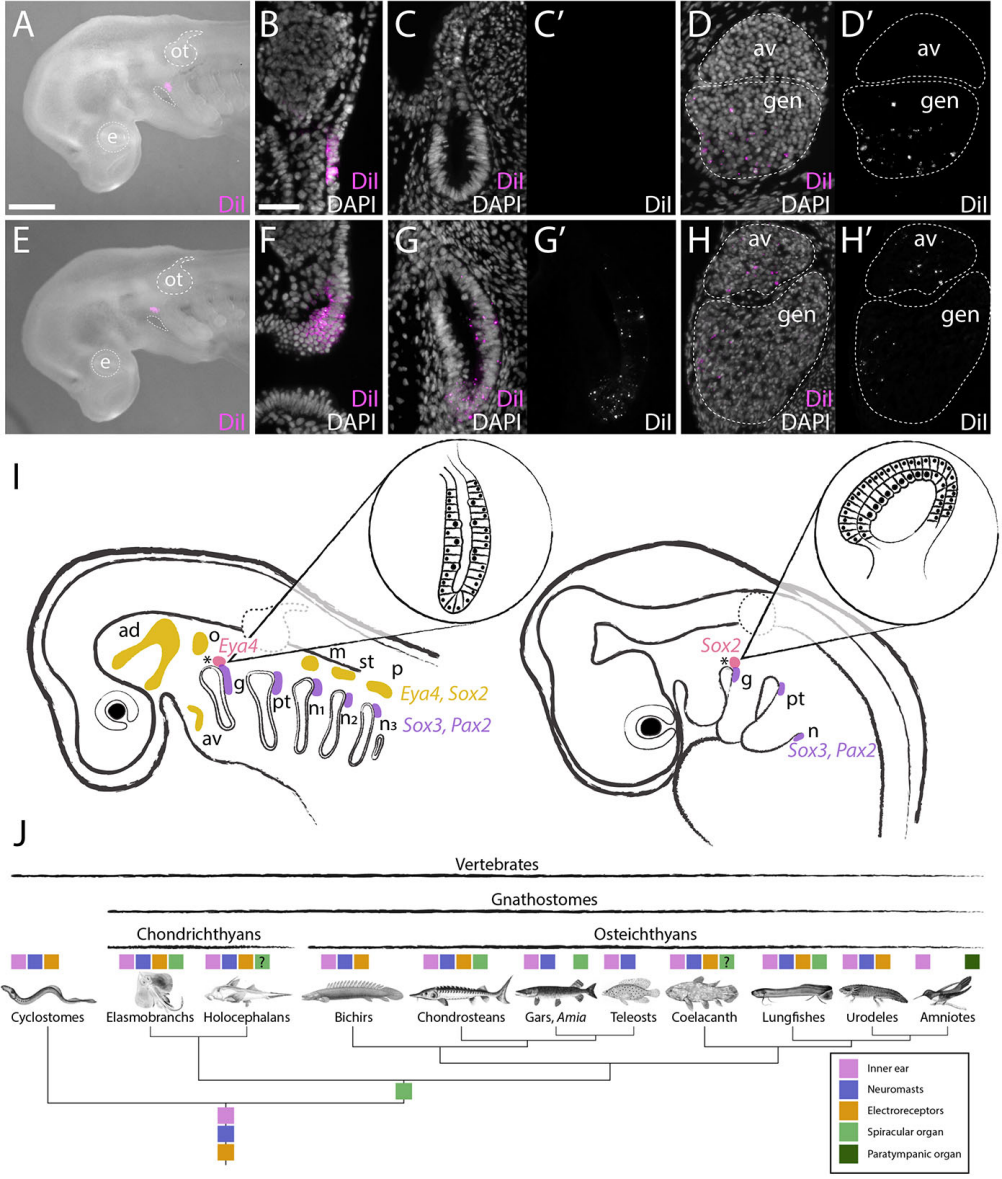
**Embryonic origin of the spiracular organ of the skate.** (A-D′) Labelling of the geniculate placode with CM-DiI at S24 (A,B) does not label the spiracular organ at S32 (C,C′), but abundant CM-DiI-positive neurons are seen in the geniculate ganglion (D,D′). (E-H′) Labelling of placodal ectoderm dorsal to the geniculate placode (i.e., the putative SpO placode) with CM-DiI at S24 (E,F) results in abundant CM-DiI-positive cells within the spiracular organ (G,G′), as well as CM-DiI-positive neurons within the geniculate and anteroventral lateral line ganglia (H,H′). (I) Schematic summary of the location of and gene expression in the lateral line, SpO and epibranchial placodes in skate (left) and the PTO and epibranchial placodes in chicken (right). (J) Phylogenetic distribution of sensory hair cell-containing sense organs in vertebrates points to an origin of the spiracular organ along the jawed vertebrate stem and its independence from the lateral line system. ad, anterodorsal lateral line placode; av, anteroventral lateral line placode or ganglion; e, eye; g, geniculate placode; gen, geniculate ganglion; m, middle lateral line placode; n, nodose placode; n_1-3_, nodose placodes; o, otic lateral line placode; ot, otic vesicle; p, posterior lateral line placode; pt, petrosal placode; st, supratemporal lateral line placode. Scale bars: 500 µm in A,E; 25 µm in B-D′,F-H′.

To fate-map the skate SpO directly, we focally labelled ectodermal domains in the dorsal region of the second (hyoid) pharyngeal arch with the lipophilic dye CM-DiI at S24. At S32, when the SpO and cranial sensory ganglia are fully developed (Fig. 2A), embryos were analysed histologically for the presence and distribution of CM-DiI-labelled cells in the SpO and cranial sensory ganglia. In all embryos in which the geniculate placode was targeted at S24 (Fig. 4A,B), CM-DiI-labelled cells were recovered only in the geniculate ganglion at S32 (*n*=6/6), but not in the SpO (*n*=0/6; Fig. 4C-D′; Fig. S3A). In all embryos in which the putative SpO placode was targeted at S24, abundant CM-DiI-labelled cells were recovered throughout the SpO (*n*=12/12; Fig. 4E,F; Fig. S3A). CM-DiI-labelled cells were also recovered in most cases in the geniculate (*n*=11/12) and anteroventral lateral line ganglia (*n*=10/12; Fig. 4G-H′; Fig. S3A). As described earlier, both successful cases of retrograde CM-DiI-labelling of the SpO at S32 identified multiple CM-DiI-positive putative SpO afferent neurons within the geniculate ganglion (Fig. 2M-N′). These experiments resolve the embryonic origin of the skate SpO from a domain of neurogenic placodal ectoderm that is anatomically equivalent to the avian PTO placode (i.e., lies immediately dorsal to the geniculate placode).

To confirm the developmental independence of the skate SpO from the lateral line system, we labelled the nearest lateral line placode (presumed to be the neurogenic pole of the anteroventral lateral line placode, as described above) with CM-DiI. We recovered no CM-DiI-labelled cells in the SpO of these embryos (*n*=0/7; Fig. S3A), although all had CM-DiI-labelled cells in their geniculate, anteroventral lateral line, anterodorsal lateral line and vestibuloacoustic ganglia (*n*=7/7; Fig. S3A). We also note that in half of the SpO placode-targeted embryos, CM-DiI-positive cells were also present in the vestibuloacoustic and anterodorsal lateral line ganglia (*n*=6/12; Fig. S3A,B,D), which form a separate ganglionic complex (Fig. 2A). The neurons of these ganglia derive from the otic vesicle and the anterodorsal plus otic lateral line placodes, respectively (Landacre, 1916; Norris and Hughes, 2020b; O'Neill et al., 2007). At S24, the otic vesicle and anterodorsal lateral line placodes are sufficiently far dorsal to the SpO placode to rule out their accidental co-labelling (compare Fig. 4E with Fig. 3C,D; see also Fig. S3C,D for additional examples of labelling immediately after injection). This suggests that, in most of these embryos, initial placodal labelling may have contaminated some mesenchyme that contributes to the vestibuloacoustic/anterodorsal lateral line ganglionic complex. We were unable to identify the otic lateral line placode at S24 in the little skate. In the shark *Scyliorhinus canicula*, the otic lateral line placode was tentatively identified by O'Neill et al. (2007) at S26 as a circular patch of ectoderm projecting from the caudoventral border of the *Eya4*-positive anterodorsal lateral line placode. This was consistent with the position of the otic lateral line ganglion at the ‘extreme posterior border’ of the anterodorsal lateral line ganglion in the shark *Squalus acanthias* (Norris and Hughes, 1920b). According to Norris and Hughes (1920b), the otic lateral line ganglion supplies the spiracular organ, in addition to the first five or six neuromasts of the main trunk line. However, at S24 in the little skate, the anterodorsal lateral line placode, with its supraorbital and infraorbital extensions, lies fairly far dorsal and also rostral to the spiracle. In contrast, our CM-DiI fate-mapping data at this stage show that the SpO placode lies caudal to the spiracle (compare Fig. 3C,D with Fig. 4E and Fig. S3C,D) and our retrograde labelling data at S32 identified afferent neurons in the geniculate ganglion.

Overall, the anatomical conservation of the neurogenic SpO/PTO placode in cartilaginous fishes and amniotes, i.e., immediately dorsal to and contiguous with the geniculate placode, further supports the homology of the SpO and PTO (see von Bartheld and Giannessi, 2011; O'Neill et al., 2012) and confirms that the last common ancestor of jawed vertebrates possessed a SpO (Fig. 4I,J). The ancestral SpO likely had a proprioceptive function for jaw movement, as proposed for elasmobranchs and lungfishes (see Barry and Bennett, 1989; Bartsch, 1994). Furthermore, the independent loss of the SpO in several jawed anamniote lineages that have retained the mechanosensory lateral line system (bichirs, teleosts and amphibians; see Fig. 4J and von Bartheld and Giannessi, 2011; O'Neill et al., 2012), and retention of the PTO in amniotes despite loss of the lateral line system, point to an evolutionary independence of the SpO/PTO from the lateral line system. The combination of a unique sensory function relating to jaw movement, together with molecular drift from lateral line placode development, may have enabled these distinct evolutionary trajectories.

Nevertheless, the SpO most likely evolved in the lineage leading to jawed vertebrates via the modification of an existing mechanosensory lateral line organ associated with the first pharyngeal cleft. Pehrson (1949) reported that the spiracular organ in embryonic lungfishes is connected by a ‘strand’ of epithelium to a ‘neuromast primordium’ (also termed a ‘vestigial organ’) in the epidermis, which he described as the most rostral primordium of a transient ‘spiracular line’ (or ‘suprabranchial line’) dorsal to each pharyngeal cleft that disappears during development (Pehrson, 1949). A clear distinction is made between the spiracular organ itself and the ‘vestigial organs’ (neuromast primordia) of the spiracular/suprabranchial line (Pehrson, 1949). As noted by Pehrson (1949) (also see Northcutt, 1989), a ‘suprabranchial line’ of neuromasts has not been reported in any other jawed vertebrate. However, such a line is found in lampreys (Holmgren, 1942; Gelman et al., 2008) (also see Northcutt, 1989). Therefore, it seems plausible that, during jaw evolution, selection for responsiveness to jaw movements might have resulted in the modification of a ‘suprabranchial’ lateral line organ associated with the first pharyngeal cleft and its afferents, with developmental drift away from the lateral line placodes. Overall, the phylogenetic distribution of lateral line organs (electrosensory organs as well as neuromasts) and the SpO/PTO, and their impressive morphological variation across taxa, highlight the extent to which vertebrates have repeatedly modified an ancestral sensory repertoire to meet the diverse sensory challenges of their environments.

## MATERIALS AND METHODS

### Embryo collection, CM-DiI-labelling and microCT scanning

*L. erinacea* eggs were obtained from the Marine Biological Laboratory (MBL; Woods Hole, MA, USA) and maintained in a flow-through seawater system at 18°C to approximately stage (S)24 (Ballard et al., 1993; Maxwell et al., 2008; Gillis et al., 2022). All animal work complied with protocols approved by the Institutional Animal Care and Use Committee at the MBL. For manipulation, a window was cut in the egg case and the embryo removed. Embryos were anaesthetised in a solution of buffered ethyl 3-aminobenzoate methanesulfonate salt (100 mg/l; MS-222, Sigma) in seawater. For fate-mapping experiments at S24, Cell Tracker-CM-DiI (ThermoFisher), diluted 1:10 in 0.3 M sucrose from a 5 µg/µl stock in ethanol, was focally injected into the ectoderm using a pulled glass capillary needle and a Picospritzer pressure injector. Embryos were allowed to recover in fresh seawater, then replaced in their egg cases and left to develop until S32. For retrograde labelling of SpO afferent neurons at S32, the heads of fixed skate embryos were dissected to expose the inner surface of the spiracle, and Cell Tracker CM-DiI was focally microinjected into the opening of the SpO using a pulled glass capillary needle and a Picospritzer pressure injector under a dissecting microscope (Fig. S2). Heads were stored in PBS+0.02% sodium azide for 12 weeks at 4°C prior to paraffin wax embedding and sectioning. For microCT scanning, a pre-hatchling *Scyliorhinus canicula* head was stained with 0.3% (w/v) phosphotungstic acid (Sigma) in 70% ethanol and scanned using a Nikon XTEK H 225 St MicroCT scanner at the University of Cambridge Biotomography Centre.

### Histology, immunohistochemistry and *in situ* hybridisation

Following euthanasia by MS-222 overdose (1 g/l), *L. erinacea* embryos were fixed in 4% paraformaldehyde in phosphate-buffered saline (PBS) overnight at 4°C, rinsed three times in PBS and stored at 4°C in PBS with 0.01% sodium azide. For histological analysis, embryos were embedded in paraffin wax as described by Hirschberger and Gillis (2022) and sectioned at 8 µm. Masson's trichrome staining was performed using the modified protocol described by Witten and Hall (2003). Immunofluorescence with anti-parvalbumin (Merck Millipore MAB1572, mouse IgG1, 1:100) and anti-Sox2 (Abcam ab92494, rabbit monoclonal, 1:200) was performed as described by Hirschberger and Gillis (2022). Nuclei were counterstained with DAPI. Chromogenic *in situ* hybridisation was performed on 5 µm paraffin sections according to the protocol of O'Neill et al. (2012), with modifications according to Gillis et al. (2012). Whole-mount *in situ* hybridisation was performed as described by Hirschberger et al. (2021). *In situ* hybridisation chain reaction (HCR) was performed on sections according to the protocol of Choi et al. (2018), with modifications, as described by Criswell and Gillis (2020). All sequence data are accessible through NCBI under the following accession numbers: *Atoh1* (OP429207), *Eya4* (JQ425114.1), *Pax2* (OP429214), *Phox2b* (OP429208), *Pou4f1* (OP429209), *Six1* (OP429210), *Sox2* (OP429211), *Sox3* (OP429212) and *Tbx3* (OP429213).

## Supplementary Material

10.1242/develop.204767_sup1 Supplementary information
