# Supplementary material for: The skate spiracular organ develops from a unique neurogenic placode that is distinct from lateral line placodes
Source: Development. 2025 Sep 29;152(18):dev204767. doi: 10.1242/dev.204767 (PMC12516317; doi:10.1242/dev.204767)
Supplement: Supplementary information [file develop-152-204767-s1.pdf]

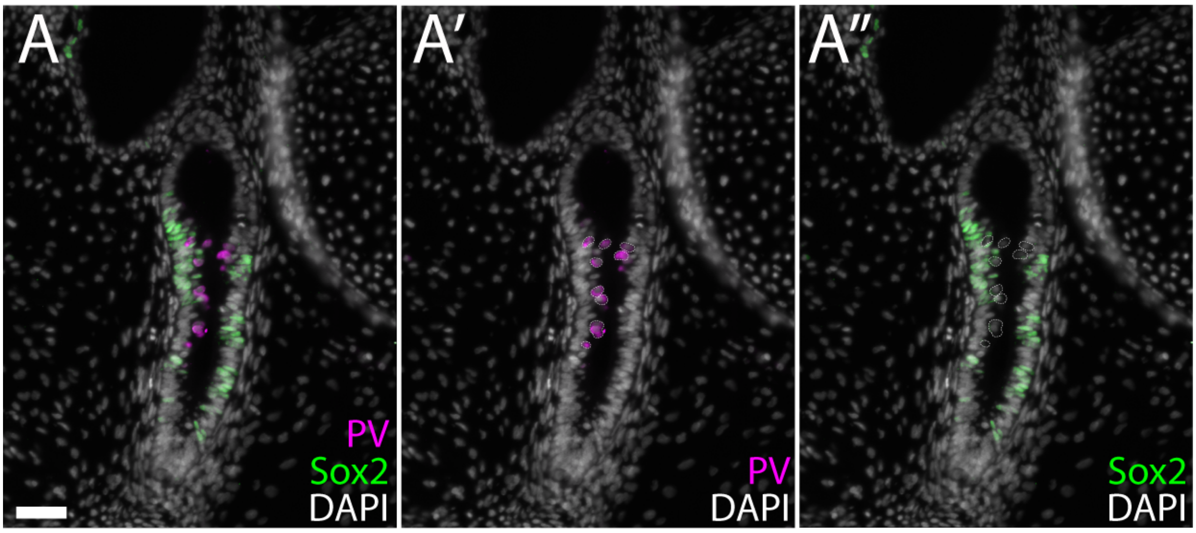


**Fig. S1. Expression of Sox2 and PV in the spiracular organ of the skate. (A)** The skate SpO expresses the hair cell marker parvalbumin (PV) and the supporting cell marker Sox2 in a mutually exclusive pattern. In **(A')** and **(A")**, sensory hair cells are outlined with a white dashed line. This image appears in Figure 2c but is shown here with imaging channels separated.

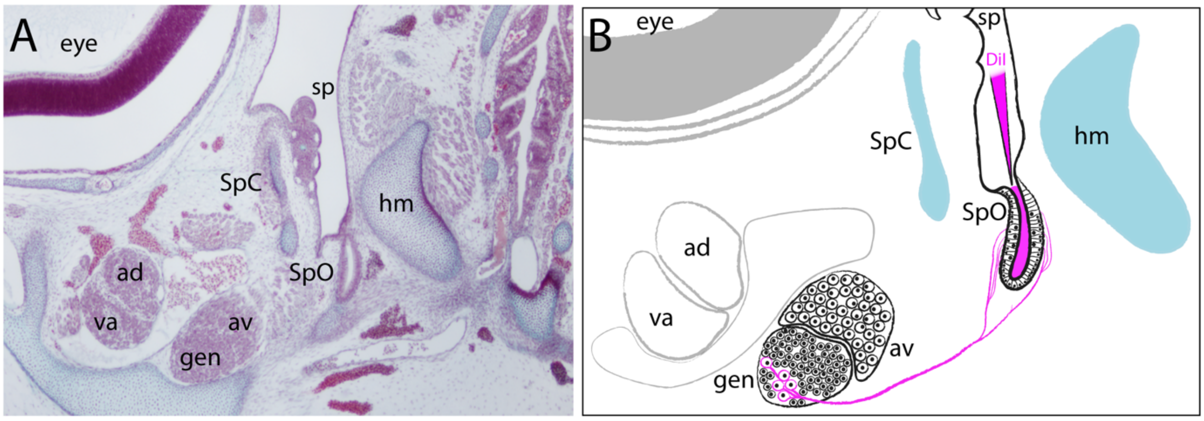


**Fig. S2. Retrograde labelling of SpO afferent neurons in the skate. (A)** Masson's trichrome-stained horizontal (frontal) section through the head of S32 skate embryo at the level of the spiracle, showing the position of the SpO relative to the eye, spiracle, cranial ganglia and spiracular and hyomandibular cartilages. A cropped version of this image appears in Figure 2a. **(B)** Schematic of the retrograde tracing strategy and outcome, drawn after the histological section in (a). CM-DiI was microinjected into the opening of the SpO of a fixed and dissected skate head at S32, and heads were stored in PBS for 12 weeks prior to histological analysis. This illustration schematises CM-DiI diffusion along axons to SpO afferent cell bodies within the geniculate ganglion. *ad*, anterodorsal lateral line ganglion; *av*, anteroventral lateral line ganglion, *gen*, geniculate ganglion; *hm*, hyomandibula; *sp*, spiracle; *SpC*, spiracular cartilage; *SpO*, spiracular organ; *va*, vestibuloacoustic ganglion.

Development • Supplementary information

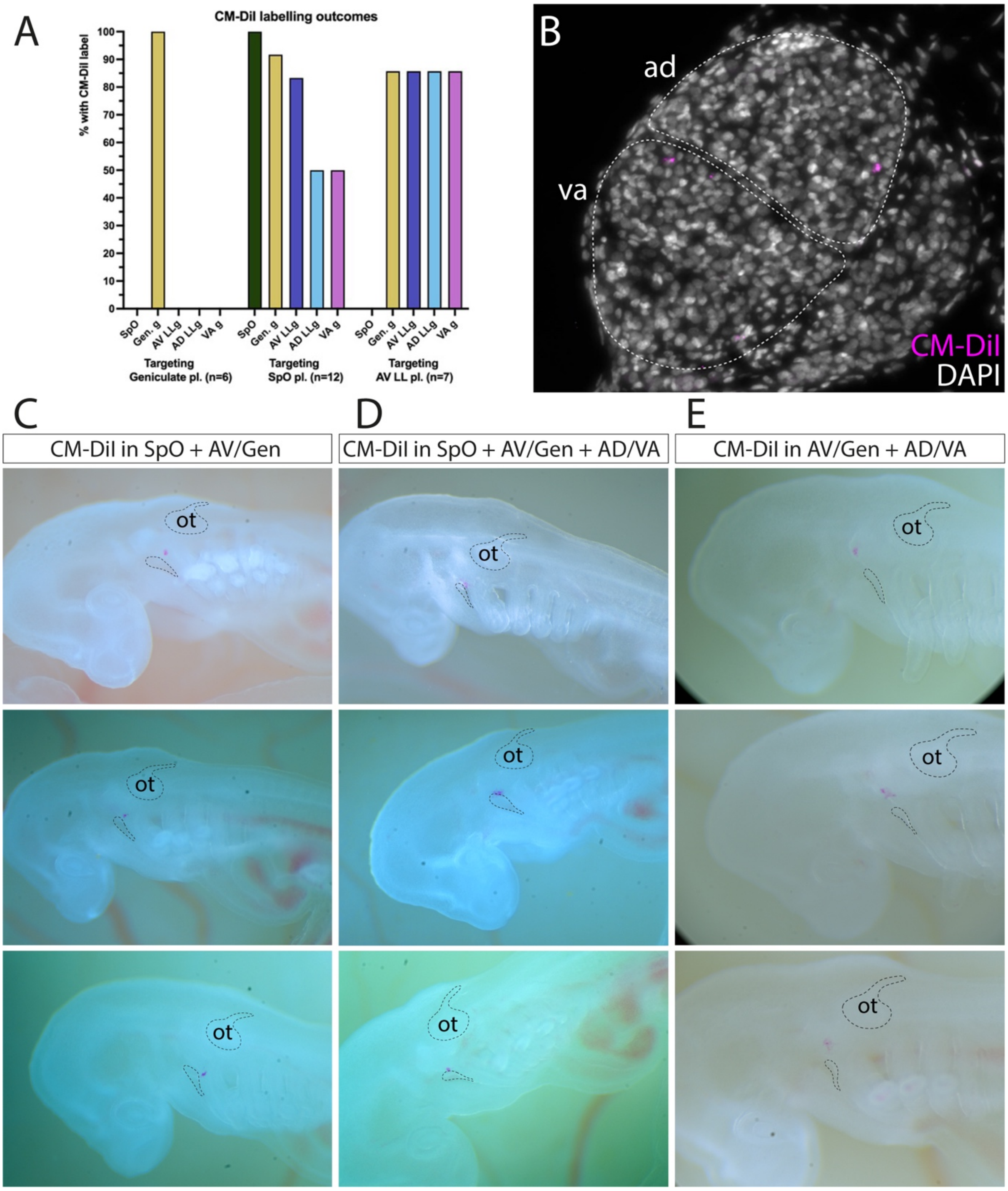
A
CM-DiI labelling outcomes
% with CM-DiI label
SpO
Gen. g
AV LLg
AD LLg
VA g
Targeting Geniculate pl. (n=6)
Targeting SpO pl. (n=12)
Targeting AV LL pl. (n=7)
B
ad
va
CM-DiI
DAPI
C
CM-DiI in SpO + AV/Gen
D
CM-DiI in SpO + AV/Gen + AD/VA
E
CM-DiI in AV/Gen + AD/VA
ot

**Fig. S3. Examples of CM-DiI targeting of the SpO and anteroventral lateral line placodes. (A)** Bar chart summarizing the proportion of embryos with CM-DiI-positive cells within the SpO and cranial sensory ganglia at S32 after targeting the geniculate, SpO or anteroventral lateral line placodes at S24. **(B)** A few CM-DiI-labelled cells were seen within the anterodorsal lateral line and vestibuloacoustic ganglia in half of the SpO placode-targeted embryos, suggesting some contamination of underlying mesenchyme at the time of placodal labelling. **(C,D)** Six skate embryos imaged immediately after targeting the SpO placode with CM-DiI at S24. In all cases, CM-DiI was recovered within the SpO, geniculate and anteroventral lateral line ganglia at S32. In three embryos (D), CM-DiI was also recovered within the anterodorsal lateral line and vestibuloacoustic ganglia at S32. **(E)** Three skate embryos imaged immediately after targeting the anteroventral lateral line placode with CM-DiI at S24. In all cases, CM-DiI was recovered within the geniculate, anteroventral lateral line, anterodorsal lateral line and vestibuloacoustic ganglia at S32**.**

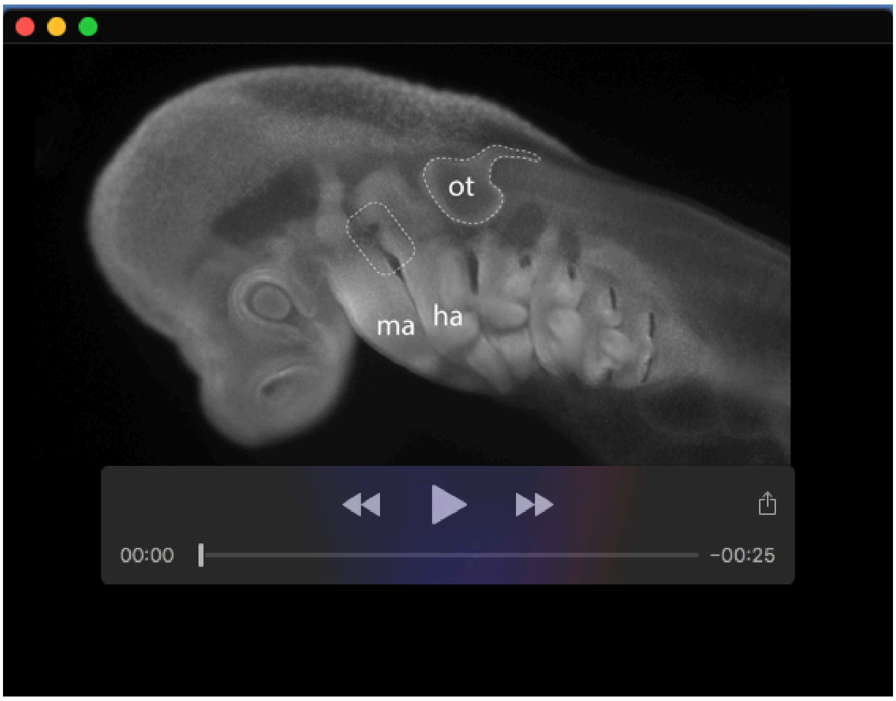


**Movie 1. The putative skate spiracular organ placode in relation to the geniculate placode and the nearest lateral line placode.** A movie created from a series of 27 adjacent transverse histological sections, in a caudal-to-rostral sequence, at the level of the second (hyoid) pharyngeal arch at S24. The series illustrates the location of the putative spiracular organ placode (*asterisk*) immediately dorsal to the geniculate placode (*g*), and its distinction from a more dorsal and rostral neurogenic placode (likely the neurogenic pole of the anteroventral lateral line placode), all three apparently giving rise to neuroblasts within a composite ganglion.

Development • Supplementary information
